# Introduction and validation of OSCAR—optimal stent choice algorithm

**DOI:** 10.1186/s42155-026-00760-1

**Published:** 2026-08-27

**Authors:** Franz Wegner, Maria-Josephina Buhné, Niclas Erben, Daniel Wulff, Erik Stahlberg, Alexander Storch, Friedrich Dünschede, Sam Mogadas, Jonas Ströder, Fabian Jacob, Malte Maria Sieren, Jörg Barkhausen, Roman Kloeckner

**Affiliations:** 1 Institute of Interventional Radiology, University of Luebeck, Ratzeburger Allee 160, Luebeck, 23538 Germany; 2 Fraunhofer Research Institution for Individualized Medical Technology and Engineering IMTE, Luebeck, Germany; 3 Institute of Radiology and Nuclear Medicine, University of Luebeck, Luebeck, Germany; 4 Institute of Robotics and Cognitive Systems, University of Luebeck, Luebeck, Germany; 5 Institute for Visual and Analytic Computing, University of Rostock, Rostock, Germany; 6 Institute of Radiology and Nuclear Medicine, Sana Hanse Hospital, Wismar, Germany; 7 Institute of Diagnostic and Interventional Radiology/Neuroradiology, Sana Hospital, Luebeck, Germany; 8 Clinical Department of Surgery and Vascular Surgery, University Hospital St. Pölten–Lilienfeld, St. Poelten, Austria; 9 Clinic of Vascular Medicine, Agaplesion Diakonie Klinikum Hamburg, Hamburg, Germany

**Keywords:** Stents, Algorithm, Interventional radiology, Decision support tool,Peripheral artery disease

## Abstract

**Purpose:**

Standardization and international guidelines for stent size selection are lacking. In this study, we introduce and validate an artificial intelligence (AI)- and image processing-supported, modular software algorithm trained on a multicentric vascular segmentation dataset that identifies stenoses, performs segmentations of stenotic vessel segments and suggests the optimal stent size for implantation.

**Material and methods:**

This retrospective multicenter study included 149 patients who underwent stent implantation for symptomatic stenoses of the common and external iliac arteries between August 2017 and July 2024. Peri-interventional angiography datasets were evaluated by four board-certified interventional radiologists. For AI-training, all relevant stenoses were annotated and segmented to reflect intended stent sizing. The segmentation criteria were consensus-defined, and all readers completed a prior training session to ensure consistency. The modular algorithm comprises components for stenosis detection, segmentation and stent parameter prediction. Following pre-training on a publicly available coronary artery dataset, the model was fine-tuned on the study-specific iliac artery dataset using leave-one-out cross-validation.

**Results:**

OSCAR detected stenoses in 84.6% of cases. The model achieved a high recall (0.89 ± 0.21), meaning that most expert-annotated stenoses were correctly identified, while a moderate precision (0.65 ± 0.28) indicated some false-positive detections. Segmentation accuracy was good (DSC 0.77 ± 0.11). Stent diameter and length predictions demonstrated mean absolute percentage errors of 0.13 ± 0.18 and 0.33 ± 0.31, respectively, comparable to expert variability.

**Conclusions:**

This proof-of-concept study demonstrates the potential of AI-assisted stent selection in vascular interventions. Furthermore, the option of a closed-loop framework promotes sustainability, reproducibility and cost-effectiveness in stent implantation procedures.

**Supplementary Information:**

The online version contains supplementary material available at https://doi.org/10.1186/s42155-026-00760-1.

## Introduction

Cardiovascular diseases (CVD) are the main cause of death in the western world [1]. The major pathophysiological aspect of most CVDs is systemic atherosclerotic transformation of the arterial vessel walls, leading to stenoses and vessel occlusions [2]. The latter can be treated by means of endovascular interventions with dedicated recanalization techniques and percutaneous transluminal balloon angioplasty (PTA) [3]. Often a PTA is followed by a stent implantation. Nowadays, there are countless stent models from different manufacturers available on the market. Choosing the optimal stent, in terms of configuration, material and size, is essential for the patient’s outcome as undersizing lead to insufficient therapy or even stent migration, whereas oversized stents can trigger neointimal hyperplasia leading to restenosis [4, 5]. Stenosis morphology and anatomical location further influence stent choice. For example, calcified lesions often require balloon-expandable stents due to their higher radial force [6].

Currently, the stent choice is a highly individualized decision, relying on operator experience. Recommendations for specific stent sizes are scarce, and internationally accepted guidelines on stent-selection do not yet exist.

In the last decade, artificial intelligence (AI) has emerged to be very promising for a broad variety of applications in vascular medicine. Especially, redundant tasks like segmentations and annotations of image data can be reliably performed by AI algorithms. Furthermore, a broad variety of preprocedural planning and decision-support tools for cardiovascular interventions became available [7]. Nevertheless, an algorithm which suggests the optimal size of the stent to implant based on DSA images is not available so far.

**Fig. 1:**
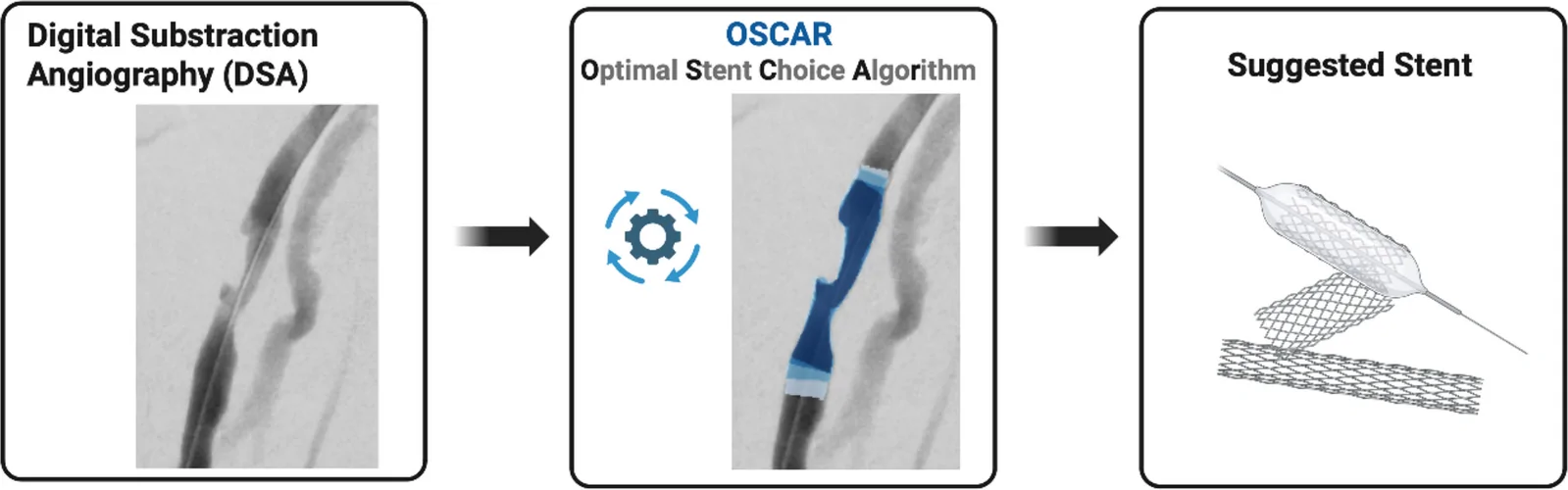
Concept-overview of the artificial intelligence (AI) and image processing-supported ‘Optimal Stent Choice Algorithm—OSCAR’. Created in BioRender. Klöckner, R. (2026)

In this study, the AI-based ‘optimal stent choice algorithm—OSCAR’ is introduced and validated. Based on a multicentric segmentation database, a modular software architecture was developed. OSCAR identifies stenoses in DSA images, performs segmentations of the stenosed vessel segment and suggests the size of the ‘optimal’ stent which should be implanted. The main principle of OSCAR is depicted in Fig. 1.

## Materials and methods

### Data acquisition

#### Study population

This retrospective study analyzed digital subtraction angiography (DSA) datasets from patients undergoing iliac artery interventions for symptomatic stenoses. Therefore, a case search has been performed in the RIS/PACS system of each study site. The target population consisted of individuals who underwent stent-implantation for iliac artery stenoses of the common iliac artery (CIA) and the external iliac artery (EIA) between August 2017 and July 2024. We focused on iliac artery interventions because they are common, typically treated with primary stenting, and usually represent isolated stenoses. The following stenoses characteristics were evaluated by an EBIR (European Board of Interventional Radiology)-certified radiologist: anatomical stenosis location, stenosis degree according to NASCET criteria, stenosis calcification (4-point grading: 0, no calcification; 1, mild calcification; 2, moderate calcification; 3, severe calcification) and stenosis configuration (unilateral/bilateral) (see Table 1). The following exclusion criteria were applied during patient selection: stent implantation for iliac artery occlusion, dissection, bleeding or arterio-ureteral fistula. Additionally, patients who underwent stent implantation using kissing technique or telescope technique, as well as those treated for vascular bypass occlusion or stenosis, were excluded. The definition of the exclusion criteria was mainly based on aspects which might influence segmentation accuracy and clarity in the early development stage of OSCAR.

**Table 1:** Detailed cohort characteristics

Variable	Cohort (*n* = 149)	
Age	Median +/− SD in years	56.9 +/− 6
Sex	Male	67.1% (100/149)
	Female	32.9% (49/149)
Stenosis location	CIA right	20.1% (30/149)
	CIA left	20.8% (31/149)
	EIA right	30.2% (45/149)
	EIA left	28.9% (43/149)
Stenosis degree	Mild (0–49%)	5.4% (8/149)
	Moderate (50–69%)	73.8% (110/149)
	Severe (70–99%)	20.8% (31/149)
Stenosis calcification	0–no calcification	22.2% (33/149)
	1–mild calcification	22.8% (34/149)
	2–moderate calcification	29.5% (44/149)
	3–severe calcification	25.5% (38/149)
Stenosis configuration	Unilateral	22.8% (34/149)
	Bilateral	77.2% (115/149)

#### Labeling workflow

The training of the AI-algorithm followed a predefined workflow. Annotations and segmentations were performed by four EBIR-certified interventional radiologists on a tablet computer (Windows Surface Pro, operating system: Windows 10 Pro). The workflow followed multiple steps. First, the image quality and complexity of stenosis configuration were graded by each reader according to a 5-point Likert scale. The evaluated statements were ‘The image quality is excellent’ and ‘The stenosis is configured simple’. Five points on the Likert scale represented ‘I fully agree’ and one point ‘I fully disagree’. The results of these evaluations were averaged for all cases and readers per center and displayed as average and standard deviation by using MATLAB (Mathworks, Natick, USA). Second, all relevant stenoses warranting treatment with stent implantation were identified by marking them with a digital pen on the tablet in the software ITK-SNAP (Penn Image Computing and Science Laboratory (PICSL), University of Pennsylvania, Philadelphia, USA). For each image, the final training annotations were selected using a heuristic approach rather than annotation fusion: the complete annotation set from the reader with the highest number of bounding boxes was chosen, and in cases of equal box counts, the annotation set with the largest mean bounding box area was used. A diameter reduction of more than 50% was defined as a stenosis requiring treatment. Finally, the treated stenosis was segmented. The entire process was supervised by a study leader, who ensured that the study was conducted in a standardized manner. To ensure inter-reader consistency, segmentation criteria were established during a consensus meeting (Table 2), and all readers completed a training session with 30 test cases. A detailed explanation of the segmentation criteria with exemplary DSA images can be found in the Supplementary Section.

**Table 2:** Segmentation criteria based on a cognitive pretesting-based consensus

Stenosis feature	Consensus-based instruction for segmentation
Length of segmentation (Suppl. Figure 1a)	The length of segmentation is chosen according to the optimal stent length with stenosis contour including the proximal and distal landing zones
Contour (Suppl. Figure 1b)	The contour is segmented according to the intraluminal contrasting
Handling of calcification (Suppl. Figure 1c)	Intraluminal calcifications should not be included into the stenosis segmentation
Handling of vascular branches (Suppl. Figure 1 d)	The over-stenting of the internal iliac artery is possible when needed from a clinical perspective
Tandem stenosis (Suppl. Figure 1e and (Suppl. Figure 1f)	If stenoses are very close to each other (< 1 cm), they are counted as one stenosis. If two or more stenosis were distant (≥ 1 cm), the case was excluded

To compare different segmentation masks per DSA image, the mean pairwise Dice Similarity Coefficient (DSC) was determined. To quantify the inter-observer labeling variability, the standard deviation (SD) of the pairwise DSC was computed (further details can be found in the Supplementary Section).

#### Algorithm architecture

**Fig. 2:**
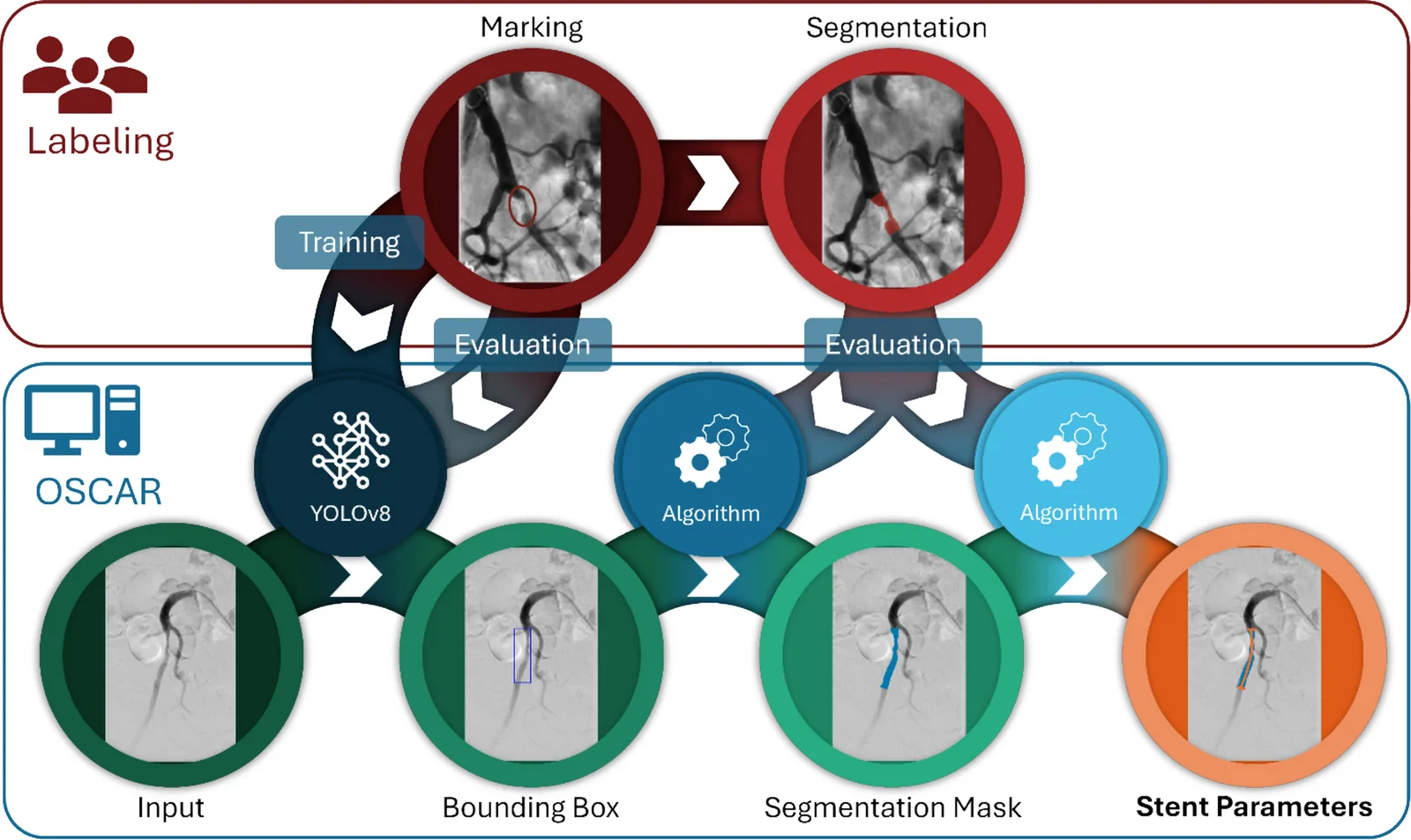
Data processing workflow of OSCAR. To generate training data for the modular algorithmic infrastructure, labeling (stenoses marking and segmentation) of DSA data was performed by four readers

OSCAR was developed in a modular style to provide understandable and explainable outcomes in every step. The pipeline consists of three independent but interoperable modules illustrated in Fig. 2 that perform (1) stenosis detection, (2) stenosis segmentation and (3) stent parameter prediction. The three modules are introduced in detail in the following sections.

#### Stenosis detection

The stenosis detection module autonomously identifies vascular narrowing in DSA images using a YOLOv8-nano–based architecture. To enhance cross-anatomical generalizability, a two-stage training strategy was implemented. The network was first pre-trained on a large publicly available coronary stenosis dataset (8325 annotated angiographic X-ray images) [8], enabling the extraction of generic stenotic features. It was then fine-tuned on the study-specific iliac artery dataset, reflecting the intended application domain. Because coronary and iliac anatomies differ substantially—small, dynamic, high-contrast coronary lesions versus larger, more static iliac stenoses—preprocessing workflows were adapted. Coronary images were cropped and resized to emphasize lesion regions, whereas iliac images underwent adaptive histogram equalization to improve contrast. Model optimization followed a progressive scheme: coronary pre-training, then iliac fine-tuning with leave-one-out cross-validation at the patient-level. Data augmentations including flipping, rotation, translation, mosaic composition and scaling were incorporated to simulate anatomical and acquisition variability and improve generalizability. Evaluation of the stenosis detection module followed standard object detection metrics: Precision (proportion of predicted stenoses that were truly present), Recall (proportion of actual stenoses that were successfully detected), mAP@50 (mean average precision at an overlap threshold (IoU) of 50%), mAP@50–95 (same measure averaged across stricter overlap thresholds (50–95%)).

#### Stenosis segmentation

**Fig. 3:**
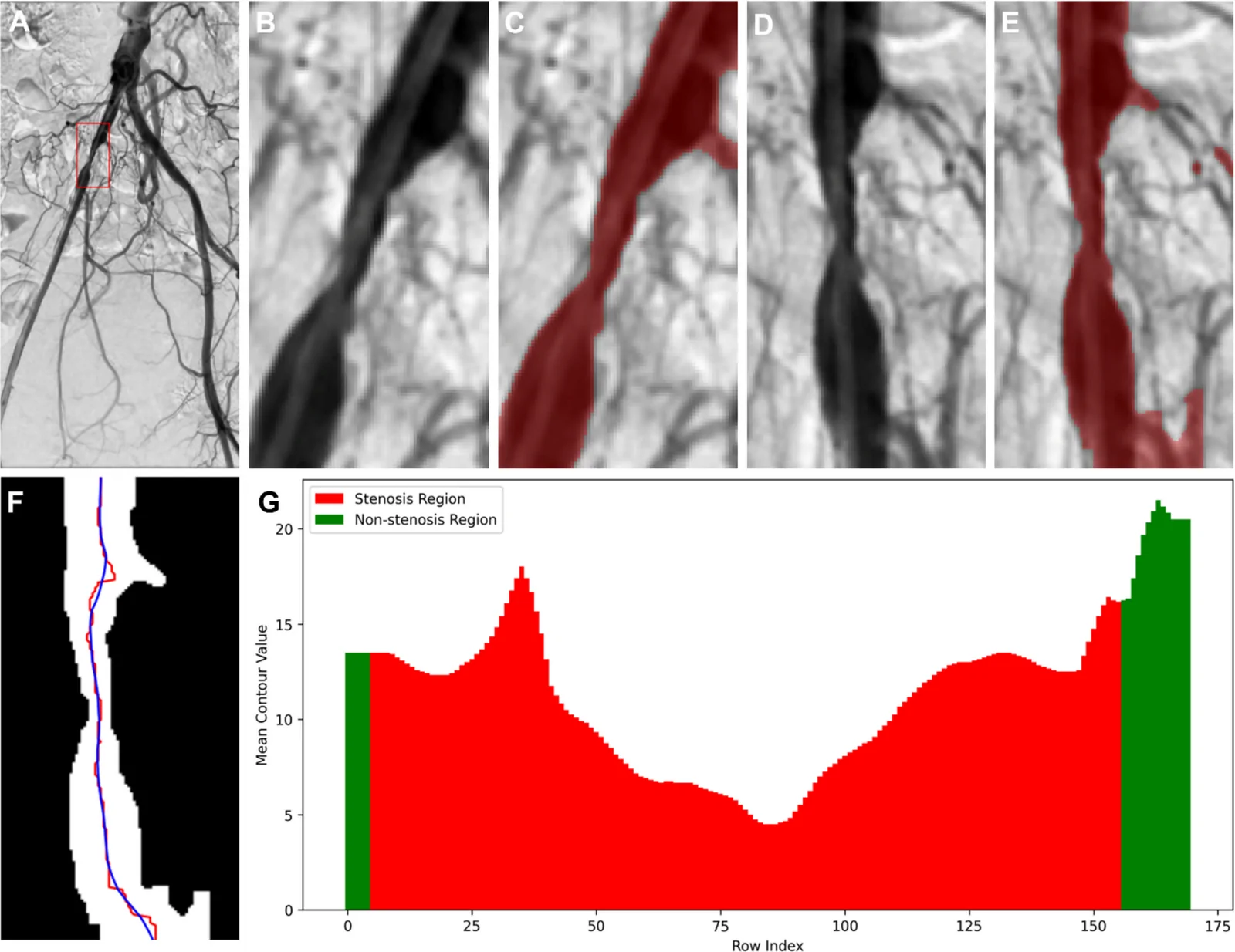
Overview of the image processing from stenosis detection to the determination of the stent parameters based on automated stenoses segmentation and analyses

In the OSCAR framework, the segmentation module isolates pathological vascular alterations with high spatial precision. Using the YOLO-derived bounding box, the pipeline integrates classical preprocessing with iterative refinement steps. The angiographic input image is first Gaussian-smoothed to suppress background noise (Fig. 3A), then cropped to the detected region to increase the lesion-to-background pixel ratio (Fig. 3B). An initial segmentation is generated via intensity thresholding (Fig. 3C), supported by standardized DSA contrast preprocessing. Morphological closing reduces local discontinuities, and the resulting mask serves as the initialization for GrabCut [9], yielding a cleaner vascular contour. To standardize geometric assessment, the segmented structure undergoes principal component analysis to determine its dominant orientation. The image is rotated to align the lesion vertically, and the bounding box is expanded by 20% to ensure full coverage (Fig. 3D). Segmentation (smoothing, thresholding, morphology, GrabCut) is repeated on this aligned representation to improve consistency in diameter and length extraction (Fig. 3E). A final watershed-based refinement [10] suppresses peripheral artifacts and preserves the core lesion geometry. Segmentation accuracy was assessed against multiple expert-derived reference masks using pairwise metrics, including DSC, precision, recall and F1-score.

#### Stent parameter prediction

Following segmentation, the OSCAR algorithm quantifies the geometric properties of the stenosis to recommend an appropriate stent diameter and length. A graph-based search identifies the vessel centerline from the proximal to distal end, automatically excluding side branches (Fig. 3F). If the segmentation is discontinuous, a vertically mirrored image is processed in parallel; both paths are fused to reconstruct a continuous centerline. This trajectory is smoothed using a moving-average filter to ensure a stable geometric reference. Using the refined centerline, left and right vessel contours are extracted, averaged and smoothed to obtain a representative stenosis profile (Fig. 3G). Lesion length is then determined from the distribution of contour values. The algorithm computes the 5th and 95th percentiles and evaluates their ratio. Ratio < 0.5: a threshold at 75% of the percentile range is applied. The binarized, smoothed contour yields stenosis boundaries at the first non-zero entries on both sides; their distance defines lesion length. Ratio ≥ 0.5: the full contour extent is taken as lesion length. The lesion diameter is derived from the 95th percentile of the contour values, offering a stable estimate that minimizes outlier influence. Further details are provided in the Supplementary Section.

## Results

### Study population

**Fig. 4:**
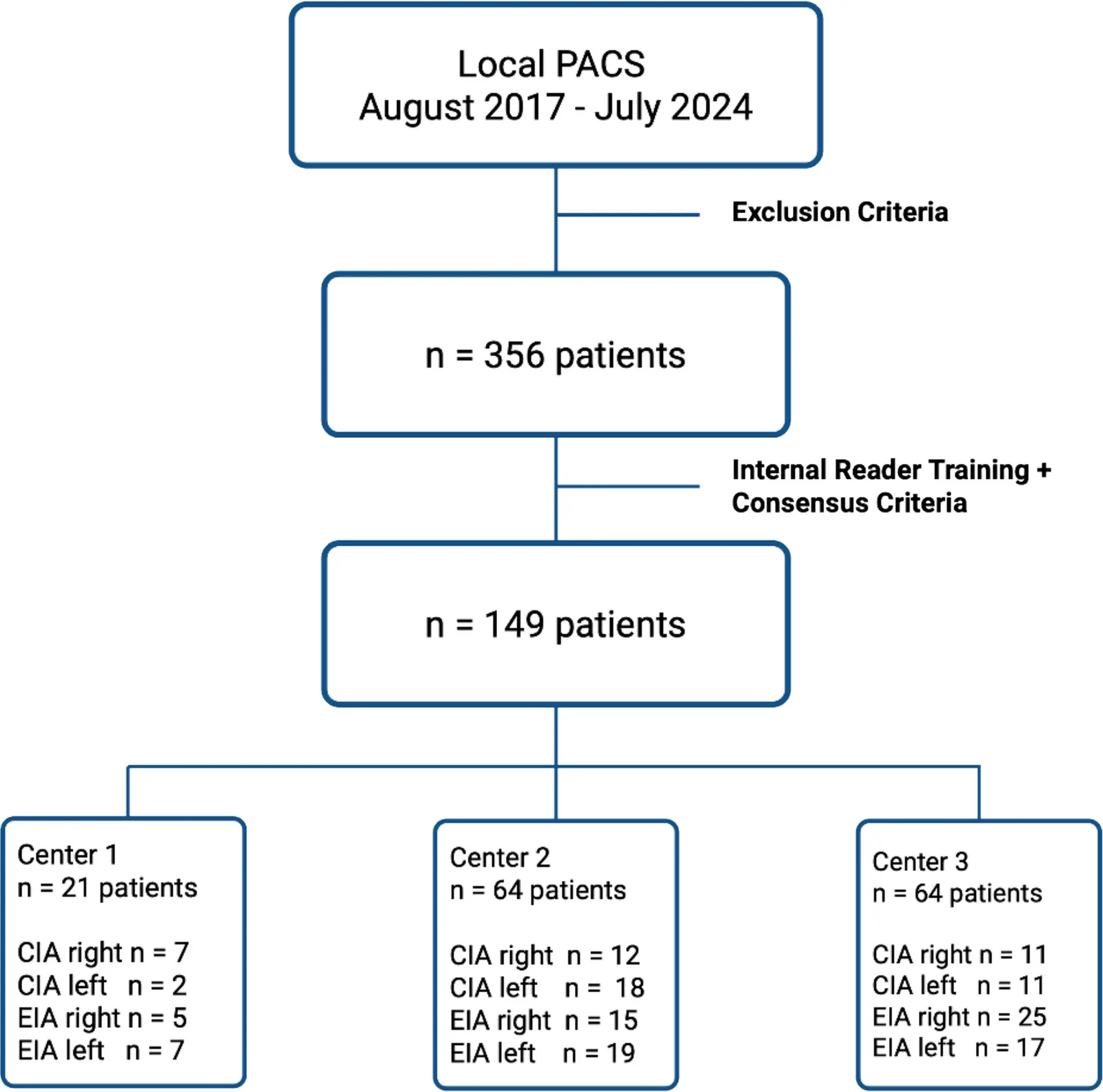
Flow chart depicting the compilation of the study cohort. Created in BioRender. Klöckner, R. (2026)

The study population consisted of 149 individuals. The median age was 56.9 +/− 6 years. Two-third of the patients were male (100/149, 67%). All patients were treated for vascular stenoses of the iliac vessels with the implantation of 30 stents in the right CIA, 31 stents in the left CIA, 45 stents in the right EIA and 43 stents in the left EIA (Fig. 4). Detailed cohort and stenoses characteristics can be found in Table 1.

**Fig. 5:**
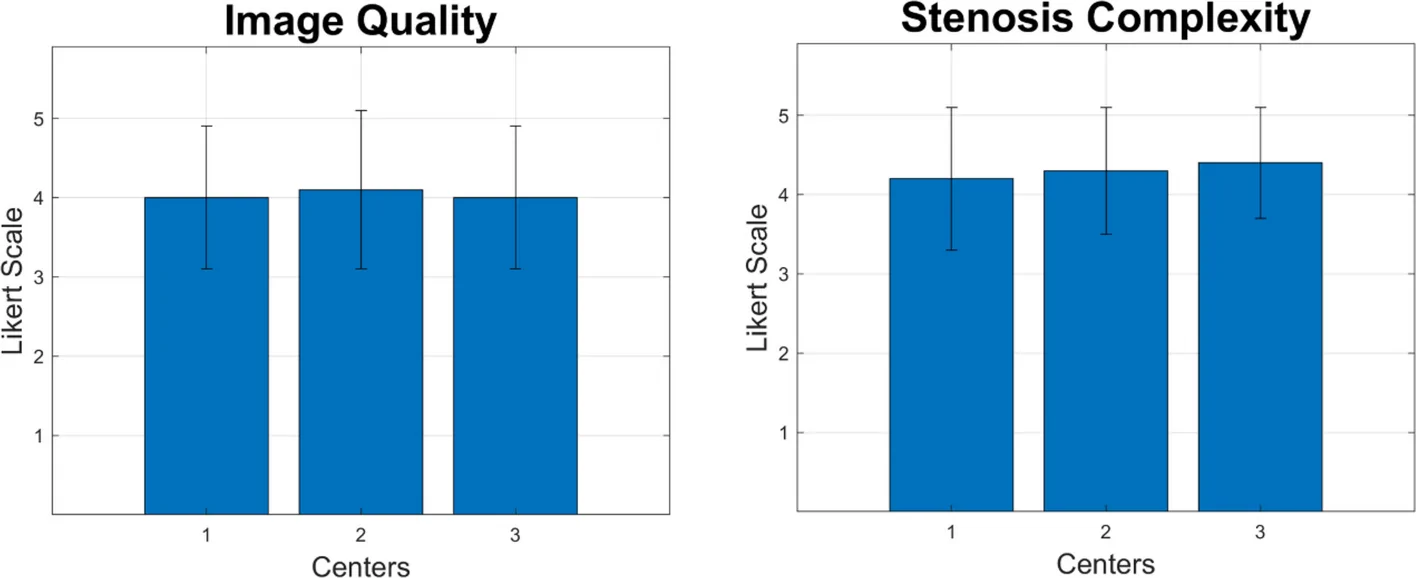
Semiquantitative evaluation of image quality and stenosis complexity by all four readers according to the three study centers. The results are displayed as average and standard deviation values

The image quality and the stenosis configuration were comparable across the three study centers (Fig. 5).

### Inter-observer variability of the segmentations

The average mean DSC determined for the dataset acquired in this study was 0.76 (range: 0.36–0.94), indicating good agreement between the expert segmentations. The average inter-observer variability was 0.11.

### Algorithm performance

#### Stenosis detection

The algorithms’ performance was assessed across 149 patients. An overview of the performance statistics is given in Table 3.

**Table 3:** Overall performance statistics (mean ± standard deviation, median (interquartile range), range)

Metric	Mean ± Std	Median (IQR)	Range
Precision	0.548 ± 0.349	0.629 (0.186–0.872)	0.000–0.994
Recall	0.751 ± 0.377	1.000 (0.500–1.000)	0.000–1.000
mAP@50	0.630 ± 0.392	0.721 (0.249–0.995)	0.000–0.995
mAP@50–95	0.276 ± 0.224	0.249 (0.062–0.418)	0.000–0.895

The algorithm successfully detected stenoses in 126 out of 149 cases (84.6%). In 23 cases (15.4%), the model did not detect any stenosis. Restricting the analysis to the 126 patients with successful detections revealed a more stable performance as shown in Table 4.

**Table 4:** Performance statistics of successful cases (mean ± standard deviation, median (interquartile range), range)

Metric	Mean ± Std	Median (IQR)	Range
Precision	0.648 ± 0.280	0.748 (0.451–0.900)	0.100–0.994
Recall	0.888 ± 0.212	1.000 (1.000–1.000)	0.333–1.000
mAP@50	0.745 ± 0.309	0.995 (0.497–0.995)	0.067–0.995
mAP@50–95	0.326 ± 0.206	0.299 (0.156–0.471)	0.018–0.895

Notably, a recall ≥ 0.8 was achieved in two-thirds of patients (66.4%), and perfect recall (1.0) was observed in 64.4% of patients.

#### Stenosis segmentation

The mean ± SD pairwise results of the stenosis segmentation measured in the evaluation were as follows: DSC: 0.77 ± 0.11 (range: 0.37–0.94), precision: 0.85 ± 0.12 (range: 0.33–0.99), recall: 0.73 ± 0.16 (range: 0.24–0.98) and F1-score: 0.77 ± 0.11 (range: 0.37–0.94).

#### Stent parameter prediction

The average pairwise mean absolute percentage error (MAPE) for the predictions were as follows: diameter: 0.13 ± 0.18 (range: 0.00–1.55), length: 0.33 ± 0.31 (range: 0.00–2.00).

For comparison, the average pairwise MAPEs between the ground truth segmentation-based stent parameters were as follows: diameter: 0.07 ± 0.06 (range: 0.00–0.26), length: 0.40 ± 0.40 (range: 0.01–3.25). The stent parameters determined from the predicted segmentation are in the min–max range of the ground truth-based stent parameters in 31% and 60% of all cases for diameter and length, respectively.

## Discussion

This proof-of-concept study shows the feasibility of AI-supported stent choice during vascular interventions. Based on a modular AI- and image processing-supported algorithm and a multi-centric training database, OSCAR can predict stent length and diameter with respect to the automatically detected stenoses in angiography with sufficient accuracy.

The spectrum of AI- and DL-based software tools for cardiovascular interventions is broad [7, 11]. On the one hand, there are tools which should improve patient selection and outcome prediction prior to procedures. On the other hand, dedicated algorithms are intended to support the interventionalist during the planning and performance of procedures. Here, navigation and decision support are the most frequently addressed aspects in currently available technologies. Many tools were developed for stenoses of coronary arteries. Langlais et al. developed ‘deepcoro’—an algorithm which can detect and assess the severity of vascular stenoses automatically [12]. ‘Ultreon’ (Abbott) is a platform which supports the clinician during OCT-guided angiographies regarding stenosis detection and stent planning. Furthermore, a calculation of the degree of stent expansion is realized by this commercial software tool, which seems to enhance the efficacy and accuracy of interventional decisions [13]. Another software platform is the ‘AVVIGO + IVUS Imaging System’ (Boston Scientific). This tool enables automated vessel measurements and real-time guidance for the placement of stents during IVUS interventions. Here, lumen overestimation is reported in a recently published in vitro study [14]. Nevertheless, to the best of our knowledge, there is currently no tool available which fully automatically suggests the optimal stent size based on DSA images for stenoses in peripheral artery disease (PAD).

Iliac stenoses and occlusions are a common manifestation of atherosclerosis/PAD [15]. In contrast to femoral lesions, iliac arteries are most often treated with primary stenting [16]. The optimal stent size has a high impact on the patient’s outcome. While oversizing triggers neointimal hyperplasia and thus potential re-stenoses [5, 17], undersized stents cause higher rates of stent thromboses [18] or even end up in dislocation. OSCAR aims to overcome the missing standardization of stent choice. Its modular architecture allows for specific training of specific tasks, which are the basis for the stent suggestion. Here, OSCAR’s YOLO-based detector shows a recall-centric profile with moderate precision. This pattern—prioritizing sensitivity, while tolerating some false positives—mirrors recent coronary-angiography detectors that also use one-stage models: for example, a YOLOv5 system reported patient-level precision ≈0.50 and recall ≈0.91 across views, emphasizing feasibility for safety screening rather than definitive ruling-in, and anchor-free/one-stage approaches (e.g. FCOS) similarly target high recall on invasive angiographies [19, 20]. In real-time and end-to-end workflows, deep learning detectors on coronary angiography have achieved robust sensitivity but still grapple with localization fidelity and false positives—particularly at stricter IoU thresholds—consistent with our lower mAP@50–95 [21, 22]. Finally, while most published benchmarks target coronary vessels, adjacent work on DSA-based vascular tasks supports the generalizability of deep models to our imaging domain, reinforcing the plausibility of OSCAR’s current performance and the rationale for domain-specific tuning [23, 24].

The core functionality of OSCAR lies in its segmentation module, which provides the key outcome parameters of stent length and stent diameter. To ensure objectivity and reproducibility, this component was evaluated using a multi-center image database with expert-generated segmentations. The inter-observer agreement reached a DSC of 0.76, indicating a reasonably high consistency among experts.

The segmentation algorithm itself achieved a DSC of 0.77, which is comparable to the inter-observer variability and thus demonstrates its reliability. Importantly, performance exceeding this level would not be expected, as the inherent variability between observers defines the upper limit of evaluation reliability. Furthermore, the DSC ranges for all observers and the algorithm were similar. Cases with lower algorithm performance were consistently associated with low observer agreement, underscoring the dependency of evaluation outcomes on annotation consistency.

Observer disagreement was most pronounced in the measurement of stenosis length, reflected by a relatively high mean absolute percentage error. This highlights the substantial observer dependency of stenosis length estimation, which complicates the reliable evaluation of OSCAR. In contrast, the annotation of stenosis diameter showed higher inter-observer agreement with OSCAR achieving a comparable performance.

From a clinical perspective, the algorithm demonstrated robust feasibility with an 84.6% overall detection success rate. The high median recall of 1.0 highlights that when the model identifies a stenosis, it frequently captures all relevant lesions. However, variability in precision demonstrates the presence of false positives in some cases. Among the 23 cases (15.4%) in which the model did not detect any stenosis, overlying bowel gas was identified as the main contributing factor. In addition, low vessel contrast, reduced image quality, and superimposition of vascular branches were frequently observed and likely contributed to the detection failure. Notably, the degree of stenosis did not differ between detected and undetected cases (63.1% vs. 61.6%), suggesting that stenosis severity itself was not a major determinant of OSCAR’s performance. However, strong sensitivity supports its potential as a reliable screening component in automated stent planning workflows. Nonetheless, moderate precision highlights the risk of false positives, which may necessitate subsequent algorithmic refinement or human oversight. Furthermore, reduced performance at stricter localization thresholds (mAP@50–95) indicates that while stenoses are generally recognized, boundary delineation remains an area for further optimization.

Although the segmentation and parameter estimation in this study were based on a non-data-driven algorithmic approach, OSCAR was able to predict lesion parameters closely aligned with expert annotations. Importantly, the sizing performance should be interpreted in the context of inter-expert variability, as expert annotations themselves frequently fell outside the corresponding ranges of other annotators, indicating that the applied evaluation criterion represents a particularly strict measure of sizing concordance. As proof-of-concept, these findings are encouraging and demonstrate the potential of OSCAR for clinical applications.

Future work should focus on enhancing stenosis segmentation through data-driven methods, such as deep learning-based approaches (e.g. YOLO, U-Net [25, 26]), and on extending parameter prediction to such frameworks.

To fulfill its clinical potential, it is necessary to integrate OSCAR as an app in an established software platform, which is used in angio-suites worldwide. In that regard, regulatory aspects must be addressed and especially included in future validation studies. In addition to its clinical value, the presented algorithm has the potential to be integrated into hospital procurement systems and thus might work as a closed-loop solution: The stents which are implanted can be automatically reordered. Furthermore, a statistical analysis can estimate the probability of specific stent types being required, allowing for more accurate inventory planning. This approach may also improve economic efficiency by reducing the number of stents that expire before implantation.

This study has several limitations which need to be discussed. The main limitation is the limited number of patient datasets which could be included in the presented work. Furthermore, we only focused on iliac lesions. Here, previous studies reveal that cross-training with angiographic images of coronary arteries shows promising performance [27]. The transfer of OSCAR to other vessel locations, especially the coronary arteries, must be evaluated in future work. Another limitation is the inter-observer variability, which we aimed to mitigate through rigorous observer training conducted in advance. Here, it must be discussed that such training could introduce a certain bias to the segmentations, which have been the basis for the training of OSCAR. Consequently, a single-blinded segmentation approach should be investigated in perspective studies. Although an independent external hold-out test set would represent the preferred strategy for assessing generalizability in large cohorts, we employed a patient-level LOOCV design to maximize the use of the limited available dataset, with each excluded patient remaining completely unseen during the respective training process. Nevertheless, future studies on larger multicenter cohorts should include dedicated external validation datasets to further confirm robustness and generalizability. Because consistent spatial calibration information was not available across all DSA datasets, the current study primarily evaluates relative geometric stenosis characterization rather than fully calibrated absolute vessel dimensions in millimeters. However, the proposed pipeline is intrinsically independent of the final unit conversion and could be extended in future work through integration of dedicated calibration strategies and multimodal validation using, for example, CT or IVUS. Additionally, the current pipeline is limited to the estimation of geometric sizing parameters and does not account for clinically relevant factors influencing stent type selection, such as calcification, vessel tortuosity, or lesion complexity, nor for differing sizing strategies between self-expanding and balloon-expandable stents. The present study excluded complex lesion and intervention scenarios such as occlusions, dissections, kissing stents and telescope techniques. Consequently, the current results may not yet generalize to the full spectrum of complex real-world iliac interventions. Additionally, we have not integrated clinical, functional and tomographic imaging data so far. Consequently, there is a need for prospective trials with multicentric data and observers in the future which assess the periprocedural and long-term clinical potential of OSCAR.

## Conclusion

In conclusion, this study proved that AI- and image processing-supported stenosis identification and stent size suggestion in vascular interventions is feasible. Furthermore, the approach comprises a closed-loop perspective, which has the potential to enhance the sustainability and economic efficacy of stent implantation procedures.

## Supplementary Information


Supplementary Material 1.

## Data Availability

The datasets and code used and/or analyzed during the current study are available from the corresponding author on reasonable request.
