# Supplementary material for: Introduction and validation of OSCAR—optimal stent choice algorithm
Source: CVIR Endovasc. 2026 Aug 27;9:108. doi: 10.1186/s42155-026-00760-1 (PMC13522298; doi:10.1186/s42155-026-00760-1)
Supplement: Supplementary file 1 [file 42155_2026_760_MOESM1_ESM.docx]

**Supplementary Section**

**Additional methodological information:**

***Inter-Observer Variability***

Two Segmentation masks and are compared by using the Dice Similarity Coefficient (DSC) defined by

To compare different segmentation masks per DSA image, the mean pairwise DSC is determined by

representing the mean similarity of all segmentation masks for a DSA image. To quantify the inter-observer labeling variability, the standard deviation (SD) of the pairwise DSC is determined.

***Stent Parameter Prediction***

The stent parameter prediction was performed based on the segmentation mask predicted by the stenosis segmentation algorithm (Sec. II.b.3). For each DSA image, the diameter and length of the required stent is determined. Since for each DSA image up to different ground truth stenosis segmentations are available, different ground truth stent parameters are available. The ground truth stent parameters are determined using the proposed algorithm as well. To evaluate the performance of the stent parameter prediction algorithm based on all ground truths, the pairwise mean absolute percentage error (MAPE) is determined by comparing the predicted stent parameters with all ground truth stent parameters by

where denotes the stent parameters.

**Suppl.Fig.1a-f: Consensus Criteria explained using example ’DO and DON’T’ cases**

1. **Stenosis feature: Length of Segmentation**

Consensus based instruction for segmentation: The length of segmentation is chosen according to the optimal stent length with stenosis contour including the proximal and distal landing zones.

**DSA-Image**	**DO**	**DON’T**

**c**

**a**

**b**

**Suppl.Fig.1a:** (a) shows a stenosis of the right AIC. (b) depicts the segmentation of the stenosed segment according to the optimal stent length with stenosis contour and proximal and distal landing zones. (c) displays the incorrect segmentation of the stenosis with focus on the stenotic segment only.

2. **Stenosis feature: Contour**

Consensus based instruction for segmentation: The contour is segmented according to the intraluminal contrasting.

**DSA-Image.**

**a**

**a**

**DO	DON’T**

**c**

**b**

**Suppl.Fig.1b:** (a) shows a stenosis of the left AIE. (b) shows the segmentation according to the intraluminal contrasting with inclusion of all bumps and scales. An incorrect segmentation with leftout of the bumps and scales is depicted in (c).

3. **Stenosis feature: Handling of calcification**

Consensus based instruction for segmentation: Intraluminal calcifications should not be included into the stenosis segmentation.

**DSA-Image**

**a**

**DO	DON’T**

**b**

**c**

**Suppl.Fig.1c:** (a) shows a stenosis of the left AIE. Calcification should not be included in the segmentation as seen in (b). An incorrect segmentation with inclusion of calcified lesions is depicted in (c).

4. **Stenosis feature: Handling of vascular branches**

Consensus based instruction for segmentation: The over-stenting of the internal iliac artery is possible when needed from a clinical perspective.

**DSA-Image	DON’T	DO**

**a**

**b**

**c**

**Suppl.Fig.1d:** (a) shows a stenosis of the AIE close to its origin. However, the AII does not need to be over-stented (b) as there is sufficient space for the landing zones (c). Conversely, the AII should be over-stented if secure placement of the stent is otherwise not possible.

5. **Stenosis feature: Tandem stenosis**

Consensus based instruction for segmentation: If stenoses are very close to each other (≤ 1cm), they are counted as one stenosis. If two or more stenosis were distant (>1cm), the case was excluded.

**Segmentation task performed:**

**DSA-Image	DO**

**b**

**a**

**Suppl.Fig.1e:** (a) shows two closely spaced stenoses. Therefore, it is classified as one stenosis and the case is segmented accordingly (b)

**Segmentation task cancelled, case to be excluded:**

**DSA-Image	Distance > 1cm	DON’T**

**a**

**c**

**b**

**Suppl.Fig.1f:** (a) shows two stenoses that are clearly distant from each other (b, distance > 1cm). This case should be ruled out, as the stenoses would be assessed as two separate stenoses. If the stenoses are segmented as a single lesion, the algorithm might be influenced regarding the optimal stent recommendation (c)
